# Active inclusion bodies of acid phosphatase PhoC: aggregation induced by GFP fusion and activities modulated by linker flexibility

**DOI:** 10.1186/1475-2859-12-25

**Published:** 2013-03-14

**Authors:** Ziliang Huang, Chong Zhang, Shuo Chen, Fengchun Ye, Xin-Hui Xing

**Affiliations:** 1 Key Laboratory for Industrial Biocatalysis, Ministry of Education, Department of Chemical Engineering, Tsinghua University, Beijing, 100084, China; 2 Current address: Department of Chemistry and Biotechnology, School of Engineering, The University of Tokyo, 7-3-1 Hongo, Bunkyo-ku, Tokyo, 113-8656, Japan

**Keywords:** Active inclusion bodies, Acid phosphatase, Green fluorescent protein, Linker flexibility, Linker engineering

## Abstract

**Background:**

Biologically active inclusion bodies (IBs) have gained much attention in recent years. Fusion with IB-inducing partner has been shown to be an efficient strategy for generating active IBs. To make full use of the advantages of active IBs, one of the key issues will be to improve the activity yield of IBs when expressed in cells, which would need more choices on IB-inducing fusion partners and approaches for engineering IBs. Green fluorescent protein (GFP) has been reported to aggregate when overexpressed, but GFP fusion has not been considered as an IB-inducing approach for these fusion proteins so far. In addition, the role of linker in fusion proteins has been shown to be important for protein characteristics, yet impact of linker on active IBs has never been reported.

**Results:**

Here we report that by fusing GFP and acid phosphatase PhoC via a linker region, the resultant PhoC-GFPs were expressed largely as IBs. These IBs show high levels of specific fluorescence and specific PhoC activities (phosphatase and phosphotransferase), and can account for up to over 80% of the total PhoC activities in the cells. We further demonstrated that the aggregation of GFP moiety in the fusion protein plays an essential role in the formation of PhoC-GFP IBs. In addition, PhoC-GFP IBs with linkers of different flexibility were found to exhibit different levels of activities and ratios in the cells, suggesting that the linker region can be utilized to manipulate the characteristics of active IBs.

**Conclusions:**

Our results show that active IBs of PhoC can be generated by GFP fusion, demonstrating for the first time the potential of GFP fusion to induce active IB formation of another soluble protein. We also show that the linker sequence in PhoC-GFP fusion proteins plays an important role on the regulation of IB characteristics, providing an alternative and important approach for engineering of active IBs with the goal of obtaining high activity yield of IBs.

## Background

Inclusion bodies (IBs) are nuclear or cytoplasmic aggregates of stainable substances, usually proteins. Although IBs are conventionally considered to be misfolded protein aggregates that are dysfunctional and undesirable, recent studies have revealed that certain IBs, known as non-classical or active IBs, consist of correctly folded protein components and their biological activities can be comparable with those of the soluble proteins [1-5]. Compared to soluble proteins, active IBs have shown unique advantages, such as easy purification and separation, high stability and high robustness in applications including immobilized biocatalysis, bioassays, biomaterials, etc. [6-8]. However, one of the key issues for making full use of the advantages of active IBs in industrial applications will be to improve the activity and ratio of IBs among the total expressed target proteins.

With increasing concerns to generate active IBs for industrially important enzymes, various approaches have been extensively studied, which can generally be summarized to three categories: optimization of culture and expression conditions (e.g., induction regime, salt concentration, temperature, aeration and promoter selection) [2,4,9], mutation of target proteins by truncation or point mutation [9-12], and construction of fusion proteins with partners that can induce IB formation [1,3,5,13,14]. The former two approaches, namely expression optimization and mutation introduction, are strongly peptide- or protein-specific [9,15], while the latter fusion protein approach is more universal and practical, providing there are suitable fusion partners that can effectively induce the formation of active IBs. Although several examples of IB-inducing fusion partners have been reported, such as *Clostridium cellulovorans* cellulose-binding module [16], foot-and-mouth disease virus capsid protein VP1 [1], and a maltose-binding protein mutant MalE31 [13], the choices are still very limited, hindering the development and application of active IBs.

On the other hand, to obtain active IBs with favorable characteristics (e.g., activity, solubility and ratio when produced in cells), design of fusion protein such as the selection of linker is another important issue although rarely reported. The linker sequence has been shown to impact fusion protein characteristics variously, depending on, for instance, its flexibility [17-20]. However, these linker effects have been studied exclusively for soluble proteins [17,19-21]. For aggregated proteins, where protein molecules are in a much more crowded environment than soluble proteins [1,5,22], the effects of linker can be expected to be more significant, yet rarely studied. These insights establish a compelling rationale to target linker sequence for the regulation of the characteristics of active IBs.

Acid phosphatase PhoC, which can be found in various bacteria, is a well-known biocatalyst with important application in food industry to produce inosine-5^′^-monophosphate, a flavor potentiator used in various foods [23,24]. PhoC is found to be quite a stable enzyme, thus if this enzyme can be expressed as active IBs, the enzymatic bioprocess can be more efficient by repeated use of PhoC, since the recovery of IBs is more feasible compared with soluble enzyme. However, PhoC has been reported to be soluble when overexpressed in *Escherichia coli*[23]. Previously, we have constructed the fusion protein of PhoC (from *Enterobacter aerogenes*) and green fluorescent protein (GFP), which was unexpectedly found to be expressed as IBs with fluorescence in *E. coli* cells [22,25].

Although GFP itself has been reported to form IBs [9,26,27], the extent depends largely on the culture conditions [5,28-30]. Under conditions where GFP alone can be expressed as soluble protein, GFP fusion with other proteins has not been considered as an IB-inducing factor contributing to the aggregation of the fusion protein so far [1,5]. Thus this interesting observation has implied the potentials of GFP-inducing PhoC-GFP active IBs and the necessity of studying the characteristics and mechanism of PhoC-GFP IBs expressed in *E. coli* cells, which may open a new way for the application of PhoC catalysis as active IBs.

In this report, we systematically examined the activities of PhoC-GFP IBs expressed in *E. coli* cells, namely the fluorescence of GFP and the phosphatase/phosphotransferase activities of PhoC. We furthermore proved the existence of aggregation of GFP moieties in IBs through non-denaturing solubilization experiments, demonstrating the key role of GFP in the active IB formation. Finally, we tried to exploit the linker sequence between GFP and PhoC domains as an approach to improve the activity levels and ratios of PhoC-GFP IBs expressed in the cells.

To the best of our knowledge, our study here represents the first example to utilize GFP fusion to induce active IB formation of another soluble protein and thereby demonstrates the potential of GFP as a novel IB-inducing fusion partner. In addition, the modulation of IB activities by linker flexibility, which is also reported for the first time here, provides an alternative and important way to engineer active fusion IBs for desired catalytic performances.

## Results

### PhoC-F-GFP fusion protein is expressed largely as IBs

**Figure 1:**
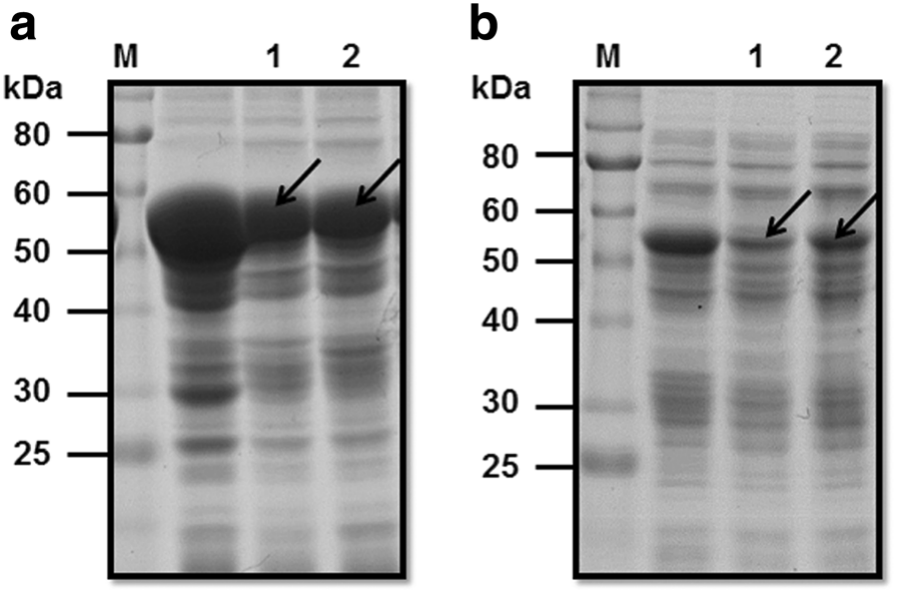
**SDS-PAGE of cell crude extracts of *E. coli *expressing PhoC-GFP fusions. a**, insoluble fractions of *E. coli* expressing PhoC-GFP fusions. M, molecular weight standard; 1, PhoC-F-GFP (F refers to the flexible linker, (GGGGS)_5_); 2, PhoC-R-GFP (R refers to the rigid linker, (EAAAK)_5_). **b**, soluble fractions of *E. coli* expressing PhoC-GFP fusions. M, molecular weight standard; 1, PhoC-F-GFP; 2, PhoC-R-GFP. Target protein bands are indicated by arrow. Lanes without number are not used in this study.

PhoC was fused to the N-terminus of GFP via a flexible linker (GGGGS)_5_ to form fusion protein PhoC-F-GFP (F refers to the flexible linker). SDS-PAGE analysis showed that the fusion protein was expressed largely as insoluble IBs in *E. coli* (Figure 1). Although efforts were made towards soluble expression through optimization of the culture conditions (e.g., culture temperature, IPTG concentration, and culture time) (data not shown), PhoC-F-GFP was still largely expressed as IBs, indicating the strong propensity for IB formation.

**Figure 2:**
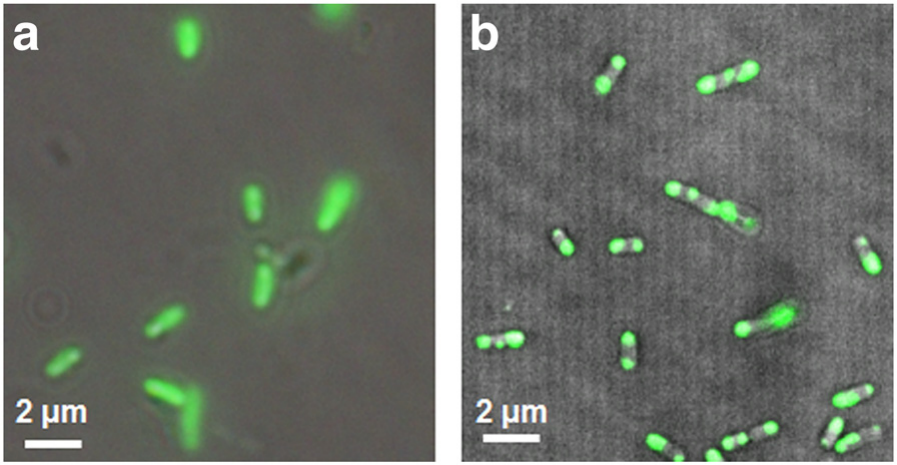
**Fluorescence microscopy images of *E. coli *cells expressing GFP (a) and PhoC-GFP (b).** The merged images of the fluorescent micrographs and the differential interference contrast micrographs are shown.

Fluorescence microscopy was also used to confirm the existence of the PhoC-F-GFP IBs in the cells (Figure 2). Particles of protein aggregates were apparently observed in *E. coli* cells expressing PhoC-F-GFP, while cells expressing GFP alone showed uniform fluorescence distribution over the cytoplasm, indicating the soluble expression of GFP alone under the same culture conditions. Similar morphology of GFP-containing IBs, which are generally induced by fusing GFP to an additional IB-inducing fusion partner has been well documented [1,5], which reinforces our results here.

### IBs of PhoC-F-GFP show high-level biological activities

**Figure 3:**
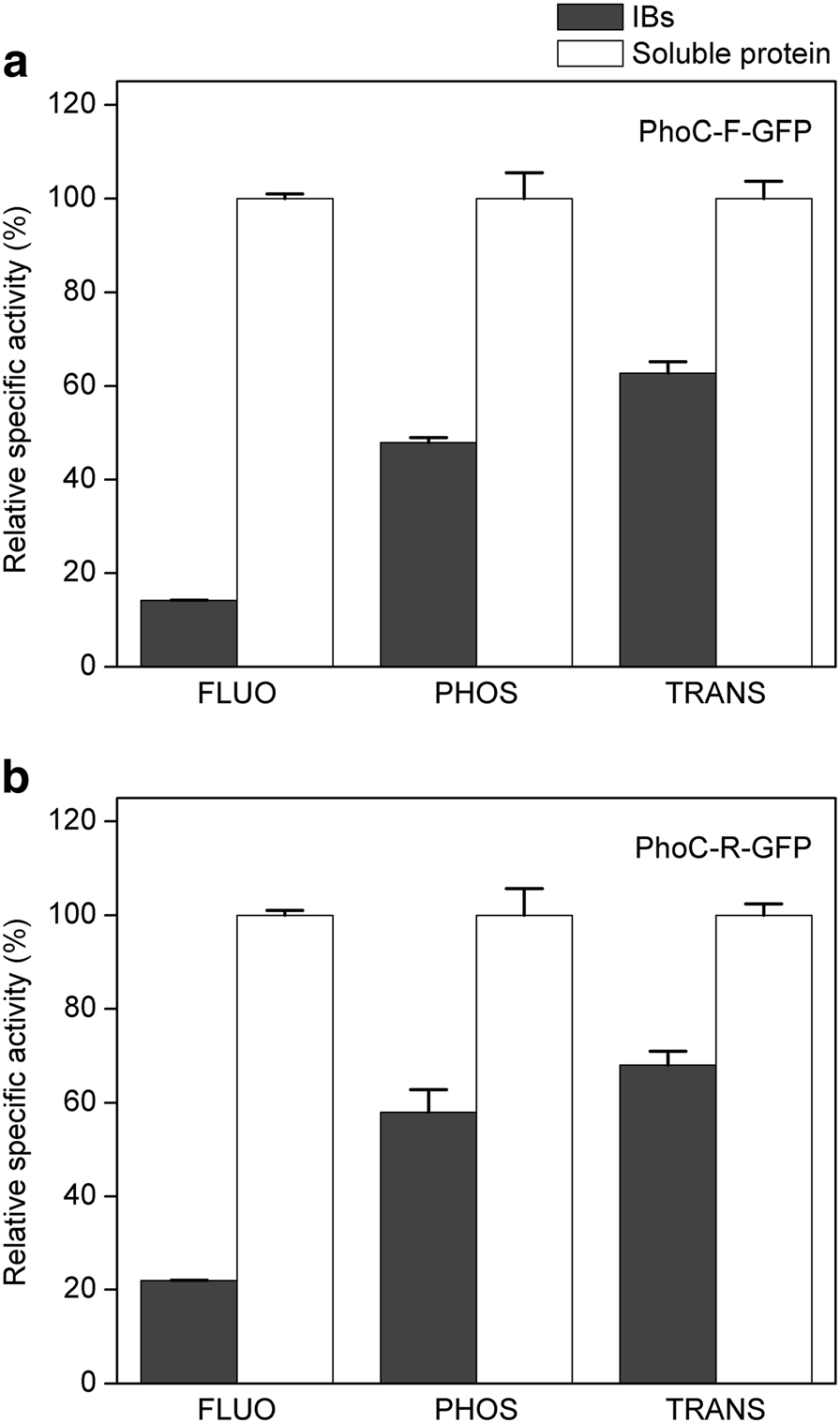
**Relative specific activities of PhoC-GFP fusions in different forms. a**, fusion protein PhoC-F-GFP. **b**, fusion protein PhoC-R-GFP. Dark grey, IBs; white, soluble protein. FLUO, fluorescence; PHOS, phosphatase activity; TRANS, phosphotransferase activity. Error bars are shown as standard deviation of three independent experiments.

**Figure 4:**
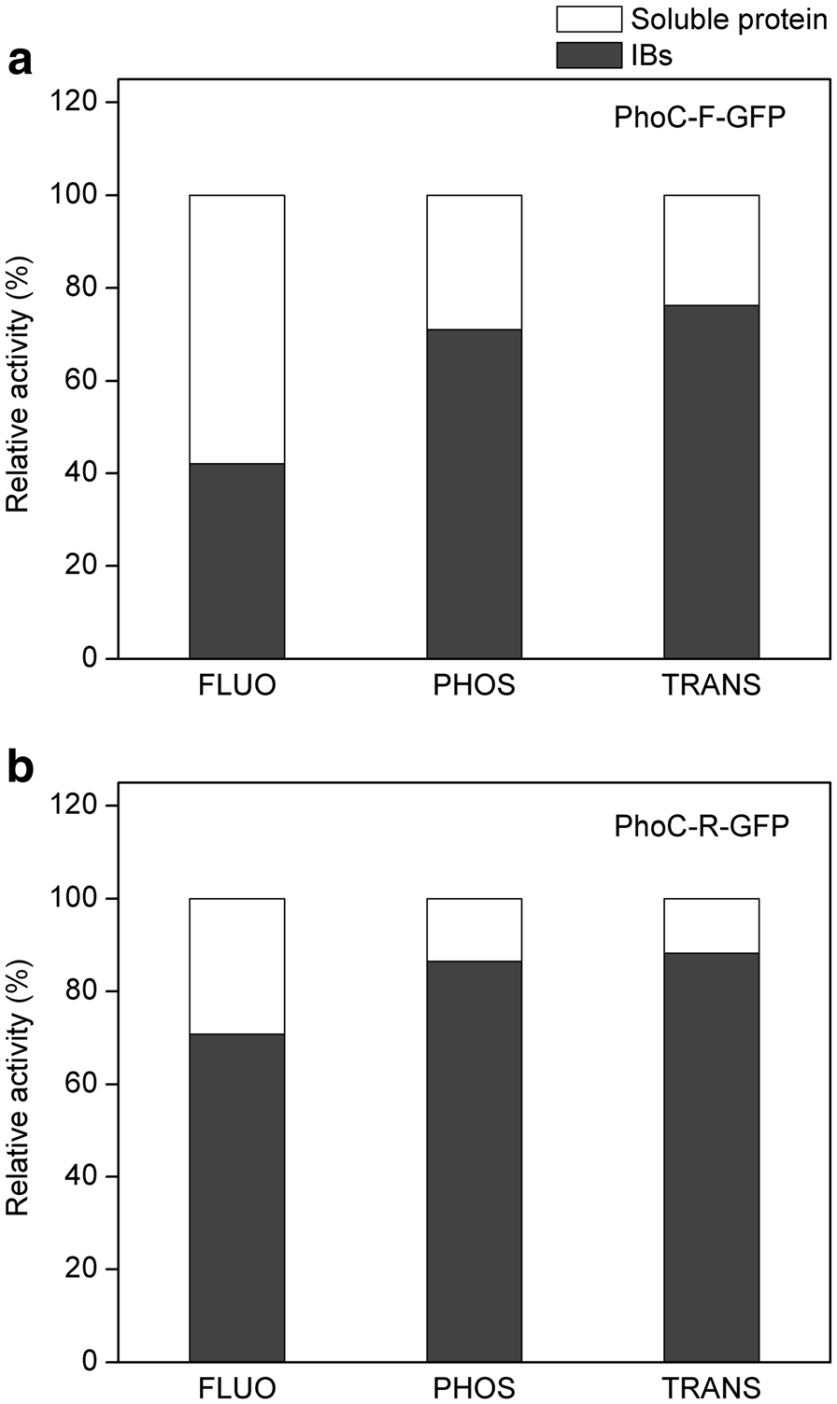
**Distribution of total activities in the soluble protein and IBs in *E. coli *cells. a**, fusion protein PhoC-F-GFP. **b**, fusion protein PhoC-R-GFP. Dark grey, IBs; white, soluble protein. FLUO, fluorescence; PHOS, phosphatase activity; TRANS, phosphotransferase activity.

The PhoC and GFP activities of PhoC-F-GFP IBs, were then systematically studied and compared with those of the soluble PhoC-F-GFP protein purified from the soluble fraction of cell crude extracts (Figure 3). Although the specific fluorescence of the IBs was 14.2% of that of the soluble fraction, the specific phosphatase activity and phosphotransferase activity of PhoC can reach up to 47.9% and 62.8% of those of the soluble fraction, respectively (Table 1), implying that the IBs are biologically active (other active IBs for reference: 19.5 − 166.4% [1]; 30.7% [11]; 77 − 120% [5]). On the other hand, considering the distribution of activities between soluble and insoluble fractions, the IBs can account for 42.1% of the total fluorescence, 71.0% of the total phosphatase activity, and 76.2% of the total phosphotransferase activity in the cells (Figure 4a), also demonstrating that the IBs are biologically active (other active IBs for reference: 95% [13]; 87.5% and 94.4% [5]). Thus our results here show that by fusing PhoC with GFP, the originally solubly expressed PhoC can be expressed as active IBs.

**Table 1:** Change of specific activities among different forms of PhoC-F-GFP

**Activity**	**Percentage of specific activity**^**1 **^**(%)**			**Solubilized IBs/IBs**^**2**^
	**Soluble protein**	**IBs**	**Solubilized IBs**	
Fluorescence	100 ± 1	14.2 ± 0.1	71.8 ± 0.2	5.06
Phosphatase	100 ± 6	47.9 ± 1.6	75.7 ± 2.2	1.58
Phosphotransferase	100 ± 4	62.8 ± 0.1	74.4 ± 2.2	1.18

^1^ The percentage of specific activity of different forms relative to the corresponding activity of the soluble protein.

^2^ Ratio of specific activity of solubilized IBs compared with IBs.

### Mechanism of IB formation revealed by non-denaturing solubilization

**Figure 5:**
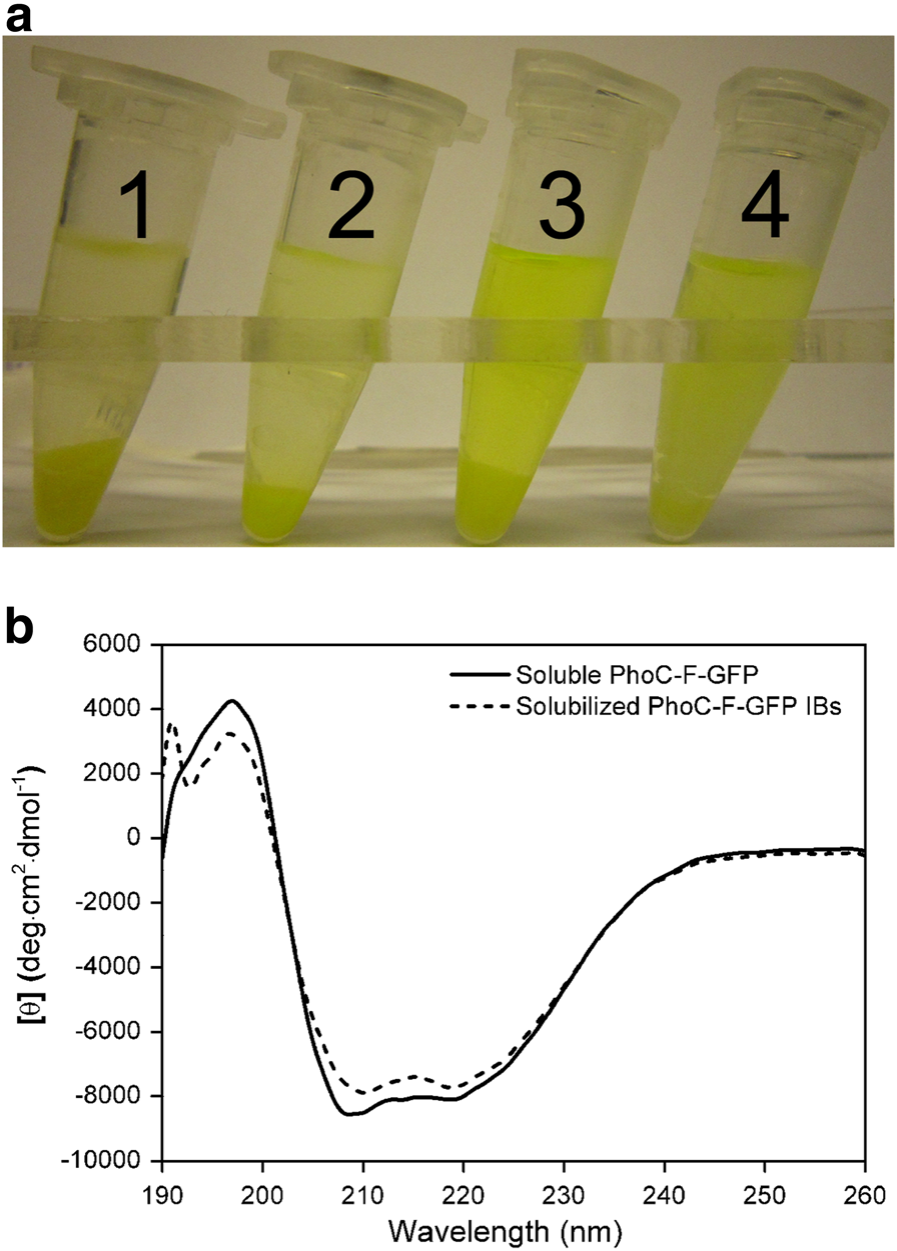
**The non-denaturing solubilization of PhoC-F-GFP IBs. a**, solubilization of PhoC-F-GFP IBs by various concentrations of arginine. Tube 1, distilled water (control); Tube 2, 0.5 M arginine; Tube 3, 1.0 M arginine; Tube 4, 2.0 M arginine. **b**, the far-UV CD spectra of PhoC-F-GFP in different forms. Solid line, soluble PhoC-F-GFP; Short dashed line, solubilized PhoC-F-GFP IBs.

As PhoC and GFP can both be solubly expressed in *E. coli* when they are not in a fusion fashion under the same culture conditions, the mechanism for the formation of PhoC-F-GFP IBs is not as clear as other active IBs containing a well-established IB-inducing fusion partner [5,13-15]. Although the addition of (His)_6_ tag might be an IB-inducing factor in some cases [9], this effect has not been observed for PhoC and GFP under our experiment conditions (data not shown), and the (His)_6_ tag has been used extensively for the purification of soluble proteins [31-35]. In addition, (His)_6_ tag is much smaller in size when compared with GFP or PhoC. Therefore (His)_6_ tag is probably not the main factor contributing to the IB formation here. This has prompted us to find out the mechanism for the formation of PhoC-F-GFP IBs. IBs can be grouped into two types, firm or loose IBs, resulting from different formation mechanisms [27,36,37]. While the firm IBs consist of unfolded protein or early-stage folding intermediates, the loose ones are mainly native proteins or late-stage folding intermediates [37]. To determine the types and the formation mechanism of the IBs obtained here, the non-denaturing solubilization of IBs was performed using the indicative solvent arginine, which is known to be only effective for loose IBs [9,26,27,37]. As shown in Figure 5a, PhoC-F-GFP IBs were effectively solubilized by arginine for all the tested concentrations (tubes 2–4), indicating that the PhoC-F-GFP IBs are loose and the protein therein maintains a near-native folding state [27]. The arginine-solubilized IBs were further subjected to circular dichroism (CD) examination. As shown in Figure 5b, the IBs showed highly similar secondary structure with that of the soluble PhoC-F-GFP [38], providing more evidence to the fact that the protein folding is near-native in the IBs [9,28].

We further examined the activities of the arginine-solubilized IBs. The GFP fluorescence after solubilization was found to improve to as much as 5-fold of that of the IBs (Table 1), which is on a comparable level with thesoluble PhoC-F-GFP, suggesting that GFP in the IBs lost much of its fluorescence activity due to extensive aggregation, as the solubilization effect of arginine is through suppression of aggregation, rather than desta-bilization of misfolded structure or facilitation of refolding [27,36,37,39]. This result is, in fact, in good consistence with the previous result that the activity of GFP is much lower in IBs than that in the soluble protein (Figure 3a). It is easy to note that the GFP moiety has lost much more activity due to its extensive aggregation, while the PhoC moiety has lost much less probably due to less aggregation degree. This analysis suggests that the aggregation of GFP, rather than that of PhoC, plays the main role in the formation of PhoC-F-GFP active IBs.

### Activities of IBs can be improved by adjusting linker flexibility

The linker sequence is well known for its role in determining the functions of fusion proteins [17-20], however its effect on the activity of the IBs of fusion proteins has not been reported before. By using different linker sequences, we speculated that the activities of PhoC-F-GFP IBs might be modulated or altered. To demonstrate this idea, a different linker sequence of (EAAAK)_5_ was used to construct the PhoC-R-GFP fusion (R refers to the rigid linker). This rigid linker, in contrast to the originally used flexible (GGGGS)_5_, is known to form a rigid α-helix conformation when expressed [17,21,40,41].

The biological activities of PhoC-R-GFP in different forms (soluble protein and IBs) were examined (Figure 3b) and compared with those of PhoC-F-GFP. Surprisingly, the relative specific activities of IBs of PhoC-R-GFP (relative to those of the soluble protein) were increased by 54.9%, 21.1%, and 8.28% for fluorescence, phosphatase and phosphotransferase respectively, compared to the values of PhoC-F-GFP (Table 2). Moreover, considering the distribution of total activities between soluble fractions and IBs, the percentage of total activities in IBs was also increased by a great extent (~16 − 68%, Figure 4). These results clearly demonstrate that a rigid linker can improve the IB activities, representing an example of controlling fusion IB activities by the linker sequence.

**Table 2:** Comparison of specific activities of PhoC-GFP fusions with different linkers

**Activity**	**Relative specific activity of IBs**^**1 **^**(%)**			**Difference in specific activity caused by linker**^**3 **^**(%)**	
	**PhoC-F-GFP**	**PhoC-R-GFP**	**Increase**^**2**^	**Soluble**	**IBs**
Fluorescence	14.2 ± 0.1	22.0 ± 0.2	54.9	15.3 (5.70 × 10^-5^)^4^	30.7 (1.76 × 10^-8^)
Phosphatase	47.9 ± 1.6	58.0 ± 1.5	21.1	8.74 (1.25 × 10^-1^)	10.4 (6.36 × 10^-3^)
Phosphotransferase	62.8 ± 0.1	68.0 ± 1.3	8.28	1.61 (7.47 × 10^-1^)	10.0 (1.15 × 10^-3^)

^1^ Calculated as the percentage of the specific activity of IBs relative to the corresponding specific activity of the soluble protein.

^2^ Increase of relative specific activity of PhoC-R-GFP compared with PhoC-F-GFP as expressed by the following equation: $\frac{\mathrm{RS}A_{\mathrm{PhoC}-R-\mathrm{GFP}}-\mathrm{RS}A_{\mathrm{PhoC}-F-\mathrm{GFP}}}{\mathrm{RS}A_{\mathrm{PhoC}-F-\mathrm{GFP}}}\times 100\%$ (RSA, relative specific activity).

^3^ Calculated as $\frac{\left|SA_{\mathrm{PhoC}-R-\mathrm{GFP}}-SA_{\mathrm{PhoC}-F-\mathrm{GFP}}\right|}{SA_{\mathrm{PhoC}-F-\mathrm{GFP}}}\times 100\%$ (SA, specific activity).

^4^*p*-values for the statistical significance between different linkers regarding specific fluorescence or activity are shown in parentheses (α = 0.05).

## Discussion

GFP has been widely used as fluorescent fusion tag in various applications [42-45]. Although several studies have reported the formation of active IBs for GFP-containing fusion proteins, there is always the involvement of another specific fusion partner rather than GFP to induce the aggregation of the target proteins [1,5]. GFP fusion has not been considered as an IB-inducing approach under conditions where GFP alone can be expressed as soluble proteins. Our present study, in contrast, demonstrated for the first time that even when GFP is fused with a soluble fusion partner, active IBs can also be formed, mainly due to the aggregation of the GFP moiety. In this context, GFP is demonstrated to have the potential as a novel IB-inducing fusion partner for some well-folded proteins.

In this study, (His)_6_ tag was used for the easy purification of PhoC-GFP fusion proteins. Although the addition of small peptide to proteins might have IB-inducing effects for some proteins [5], (His)_6_ tag has been widely used to facilitate purification, where soluble target proteins can be expressed [31-35]. More importantly, we have confirmed that the addition of (His)_6_ tag to GFP or PhoC did not lead to the formation of IBs under the same conditions, thus the addition of (His)_6_ tag to the fusion protein PhoC-GFP is not an important factor that contributes to the formation of PhoC-GFP IBs [5], but only making the purification of PhoC-GFPs much easier in this study.

The solubilization of IBs by arginine has revealed that GFP in IBs maintains near-native folding, and thus the low GFP fluorescence in IBs should be attributed to the aggregation of GFP moiety [27,36]. This aggregation has probably led to the formation of PhoC-GFP IBs. Supports for this mechanism can be found in other IB formation researches, where they showed that the intermolecular interaction between folding intermediates is the major cause for IB formation [5,13,27,46].

After arginine was removed from solubilized PhoC-GFP by dialysis, no aggregation was observed for the fusion proteins (data not shown), thus we further speculated that aggregation of GFP moiety probably resulted from the hydrophobic interactions between the hydrophobic patches exposed on the surface of folding intermediates of GFP. It has been reported that fusion can reduce the folding yield and rate of the GFP moiety [25,46,47]. Similarly, the GFP domain in PhoC-GFP fusion protein can be reasonably supposed to fold less efficiently and rapidly than non-fusion GFP, presumably prolonging the intermediate-folding time [13,46]. This also depletes the available molecular chaperones in the cell [48,49]. Taken together, all these consequently facilitate the aggregation of the folding intermediates of GFP moiety in fusion proteins [39,50,51]. For PhoC domain, due to the intrinsic folding characteristics [23,52], folding can be reasonably expected to be faster than GFP. On the other hand, as PhoC domain is translated prior to GFP domain in the fusion sequence in this study, the available folding time is longer than that of GFP [13]. Therefore, when PhoC-GFP folding-intermediates were incorporated into IB nucleus, PhoC was probably folded well and retain the native or native-like structure in the IBs. This structure is less probable to interact with other PhoC moieties to form aggregates which would harm PhoC functions [15]. This hypothesis can be supported by the fact that change in PhoC specific activities between soluble form and IB form is much smaller than that of GFP (Figure 3), suggesting similar folding state in IBs and in the soluble protein for PhoC, and no obvious aggregation in IBs.

In this study, we also exploited the linker sequence to modulate the activities of IBs for the first time. In fact, besides the improvement of activities shown in *Results*, we noticed that the effect of linker sequence is even more significant in IBs than in soluble proteins. For example, the differences in specific activities (GFP, phosphatase and phosphotransferase) of soluble fractions between PhoC-R-GFP and PhoC-F-GFP were 15.3%, 8.74%, and 1.61%, respectively, while the differences between their IBs were 30.7%, 10.4%, and 10.0%, respectively (Table 2), showing more significant effects of linker sequence for IBs than for soluble proteins. In addition, for the active IBs, the change of linker shows high statistical significance regarding the specific fluorescence, phosphatase and phosphotransferase activities (α = 0.05, Table 2), respectively. Whereas for the soluble proteins, the change of linker shows less statistical significance, which is in good agreement with our suggestion that linker would have more effect for the aggregated form proteins. In fact, studies on linker have pointed out their role in controlling the conformation of fusion proteins (e.g., the distance and orientation between domains, and folding of domains) [17-20], but mostly focusing on the study of soluble proteins. Regarding the spatial relationship of domains, on which the linker region exerts its influence, the role of linker can be expected to be comparable or even more significant in IBs than in soluble proteins, as the former is a much more crowded environment than the latter [1,5,22]. The effects of linker on aggregation of the fusion proteins is probably in the following two aspects: (i) linker sequence is believed to modulate the distance between PhoC and GFP, thus affecting the aggregation of GFP moieties; (ii) on the other hand, the linker sequence itself, which is directly linked to the target protein sequence, would possibly affect the folding of proteins, thus exposing hydrophobic regions susceptible for the aggregation of the fusion proteins. Therefore our results suggest that linker property (flexibility, length, hydrophobicity, etc.) can be a potential way to engineer IBs for desired characteristics of active IBs.

For targeted engineering of active IBs, much more effort would still be needed to study the relationship between IB characteristics and linker properties. For this purpose, a systematic study of IB variants with different linkers by using a novel linker library with widely controllable and traceable flexibility developed by our group is undergoing now, which would provide important clues for the design of IBs, and should be of general importance for their industrial applications.

The partial aggregation of the target protein would result in distribution of enzyme activities among soluble and insoluble fractions, which would probably hinder the activity yield of IBs and their application. However, our results have shown that by using linkers of different flexibility, the distribution of total activities can be altered, with over 80% PhoC activities in IBs for PhoC-R-GFP (Figure 4b). This high PhoC activities in IBs could benefit the total recovery efficiency and the reuse of enzyme by the simplified protein separation via IBs. Further optimization of linker could probably lead to even higher level and ratio of activities in IBs, which is indispensable for the bioprocess application of the active IBs.

With respect to the bioprocess application of active IBs, the stability of the active IBs and enzymatic activities is of general importance. In fact, IBs have been reported to be stable [53,54]. Bioprocess studies on some active IBs have demonstrated the feasibility of repeated use of active IBs as biocatalysts [1,55-58], indicating good stability of the active IBs and enzymatic activities. Systematic studies on the stability of active IBs in real bioprocess conditions were also reported [16,59,60]. Taken together, further elucidation of the effects of linker properties on the stability of IBs and enzymatic activities will be indispensable for application of IBs in bioprocesses.

In addition, growth conditions and medium composition are also important parameters for the formation of active IBs. To examine the stability and maintenance of the activity of IBs for bioprocess application, further study on the effects of culture conditions by using more systematic experiment design such as fed-batch cultivation should be carried out in the next step.

## Conclusions

In this study, we have successfully generated active IBs of PhoC through the fusion with GFP. To the best of our knowledge, this is the first example to utilize GFP fusion for the induction of active IBs of another soluble protein, demonstrating the potential of GFP as a novel IB-inducing fusion partner. We further improved the activity levels of PhoC-GFP IBs and their ratios when expressed in cells by changing the linker flexibility, and showed the important role of linker sequence for the characteristics of active IBs. Thus our results has provided a way to obtain active IBs with desired characteristics by engineering the linker sequence. Further research on the effects of linkers with wider range of properties is therefore of importance to improve the activity yield of IBs, which would open a new route for the industrial application of PhoC catalysis.

## Methods

### Plasmid construction

Plasmid pET-28a(+) (Novagen, Madison, WI) was em-ployed for the fusion protein expression in *E. coli* cytoplasm. DNA fragments encoding enhanced green fluorescent protein (GFP) were cloned from the plasmid pHygEGFP (Clontech, Mountain View, CA) by standard polymerase chain reaction (PCR) with primer pairs containing part of the desired linker and *Bam*HI or *Not*I (depend on linker type) and *Hin*dIII sites (GFP-R-For, 5^′^-AAATATGCGGCCGCGAAAGAAGCCGCGGCGAAAGAAGCCGCGGCGAAAATGGTGAGCAA-3^′^; GFP-F-For, 5^′^-TTAGGATCCGGTGGCGGTGGCTCGGGTGGCGGTGGCTCAATGGTGAGCAAGGGCG-3^′^; GFP-Rev, 5^′^-TCCAAGCTTTTACTTGTACAGCTCGTCCATGCCGAG-3^′^. The restriction sites are underlined). DNA fragments encoding *phoC* gene [GenBank:EU182577.1] were prepared from the genomic DNA of *Enterobacter aerogenes* IAM1183 by standard PCR and primer pairs containing part of the desired linker and *Bam*HI or *Not*I (depend on linker type) and *Eco*RI sites (PhoC-For, 5^′^-TTGAATTCTTTGCGCTGGTTCCCGCCGGCAATGA-3^′^; PhoC-F-Rev, 5^′^-TTAGGATCCACCGCCACCTGAGCCACCGCCACCCGAGCCACCGCCACCATTTCGCTGTT-3^′^; PhoC-R-Rev, 5^′^-TTATTTCGCGGCCGCTTCTTTCGCCGCGGCTTCTTTCGCCGCGGCTTCATTTCGCTGTT-3^′^). Amplified fragments were digested and ligated into pET-28a (+) between the *Eco*RI and *Hin*dIII sites to give the plasmids pET28/PhoC-R-GFP and pET28/PhoC-F-GFP. The amino acid sequences of the designed linkers were: rigid linker (used in PhoC-R-GFP), EAAAKEAAAKEAAAKEAAAKEAAAK (25 amino acids); flexible linker (used in PhoC-F-GFP), GGGGSGGGGSGGGGSGGGGSGGGGS (25 amino acids). The DNA sequences of the fusion proteins were confirmed by sequencing using a 3730xl DNA analyzer (ABI, Vernon Hills, IL).

### Protein expression and purification

For all cultivations, Luria-Bertani (LB) medium (10 g/L tryptone, 5 g/L yeast extract, 10 g/L NaCl, pH 7.5) containing 50 μg/mL kanamycin was used. *E. coli* BL21 (DE3) (Stratagene, La Jolla, CA) was transformed with the indicated plasmids and cultured on LB agar plates. For protein expression, liquid LB medium was inoculated with an overnight culture of strains harboring pET28a/PhoC-R-GFP or PhoC-F-GFP, and cultured at 37°C for 2 h. When the optical density at wavelength of 600 nm (OD_600_) reached 0.6-0.8, isopropyl β-D-1-thiogalactopyranoside (IPTG) was added to a final concentration of 0.04 mM to induce expression of the fusion protein, and cells were cultivated for another 20h at 16°C. Then the harvested cells were suspended in lysis buffer (50 mM sodium phosphate, 500 mM NaCl, 20 mM imidazole, pH 7.4),after washed with phosphate buffered saline (PBS) three times, and disrupted by ultra-sonication. The supernatant was obtained after centrifugation at 13,000 × *g* for 30min at 4°C. Fusion proteins with (His)_6_-tag were purified using HisTrap HP columns (GE Healthcare, Chalfont St Giles, BUCKS, UK) according to the manufacturer’s protocol. Fractions with sufficient target protein were collected. Protein concentration was determined by Bradford protein assay kit (Bio-Rad Laboratories, Hercules, CA) with bovine serum albumin (BSA) as the standards. Precipitated insoluble particles were suspended in lysis buffer for fluorescence measurement and PhoC activity assays after washing with PBS three times to remove residual soluble proteins. The amounts of target proteins in different fractions were determined densitometrically by sodium dodecyl sulfate-polyacrylamide gel electrophoresis (SDS-PAGE) using BSA as the standards.

### Assay of phosphotransferase activity

Phosphotransferase activity was assayed in a reaction mixture containing 100 μmol of sodium acetate buffer (pH 4.2), 40 μmol of inosine, 100 μmol of Na_4_P_2_O_7_⋅10H_2_O, and enzyme solution in a total volume of 1 mL [24]. The reaction was stopped by adding 0.2 mL of 2 N NaOH. Quantitative determination of inosine and 5^′^-IMP (inosine monophosphate) was carried out by HPLC using a HC-C18 column (4.6 × 150 mm, 5 μm, Agilent Technologies, Santa Clara, CA) with detection at 245 nm. The mobile phase was 5 mM potassium phosphate buffer (pH 2.8): methanol (95:5, v:v) and the flow rate was 1 mL/min. One unit of phosphotransferase activity was defined as the amount of enzyme that produced 1 μmol of 5^′^-IMP per min under the assay conditions.

### Assay of phosphatase activity

Phosphatase activity was assayed by monitoring the rate of hydrolysis of *p*-NPP [24]. The reaction mixture contained 100 μmol of MES-NaOH buffer (pH 6.0), 10 μmol of *p*-NPP, and the enzyme solution in 1 mL. The reaction mixture was incubated for 10 min at 30°C and stopped by adding 0.2 mL of 2N KOH. Release of *p*-nitrophenol (*p*-NP) was measured at 410 nm. One unit of phosphatase activity was defined as the amount of enzyme that produces 1 μmol of *p*-NP per min under the assay conditions.

### Non-denaturing solubilization of IBs of PhoC-GFP

The precipitated IBs were suspended in arginine solutions at various concentrations containing 50 mM Tris–HCl (pH 8.0) and 200 mM NaCl [26]. Suspensions were kept at 4°C for 12 h, followed by centrifugation at 13,000 × *g* for 30min at 4°C. For the 2.0 M arginine supernatant, solubilized PhoC-GFP was subjected to dialysis against lysis buffer before fluorescence measurement or PhoC activity assays.

### Measurement of fluorescence intensity and circular dichroism (CD) spectra

The fluorescence intensity of the fusion proteins at 512 nm of the EGFP emission peak was measured using a fluorescence spectrophotometer F-2000 (Hitachi, Japan) with 488 nm excitation at 25°C. The CD spectra of fusion proteins were measured using a π-180 CD spectrometer (PiStar, Applied Photophysics, UK) at 20°C, calibrated with PBS buffer. Spectra in the far UV (260–190 nm) were recorded in cells of 1.0-mm path length using protein concentrations of 0.2 mg/mL in PBS buffer, pH 7.4. In each case, three scans (recorded at scan rate of 15 nm/min) were averaged and corrected by subtraction of the buffer alone spectrum. The far UV spectrum was analyzed by the CDNN Program (Applied Photophysics, UK) [38] to estimate the contribution of regular secondary structure elements.

## Competing interests

The authors declare that they have no competing interests.

## Authors’ contributions

ZH designed the experiments, performed most of the experiments, and prepared the manuscript. CZ participated in its design, and supervised the experiments, and revised the manuscript. SC participated in its design, and revised the manuscript. FY performed preliminary experiments. XX conceived of the study, and participated in its design, and supervised the experiments, and revised the manuscript. All authors read and approved the final manuscript.
